# Planetary Health in Health Guidelines: Expert Workshop Report 2024

**DOI:** 10.1002/gin2.70072

**Published:** 2026-04-15

**Authors:** Pakeezah Saadat, Alina Herrmann, Bernardo Sousa‐Pinto, Charlotte T. J. Michels, Denise Thomson, Emily Senerth, Grigorios Leontiadis, Jonathan Drew, Josep M. Anto, Karolina Scahill, Klaas Miersch, Lorna Benton, Maria‐Inti Metzendorf, Rafael José Vieira, Jean Bousquet, Holger Schünemann, Thomas Piggott, GRADE Planetary Health Project Group

**Affiliations:** ^1^ Department of Health Research Methods, Evidence, and Impact McMaster University Hamilton Ontario Canada; ^2^ Institute of Global Health University Hospital and Medical Faculty, Heidelberg University Heidelberg Germany; ^3^ Institute of General Medicine University Hospital and Medical Faculty, Cologne University Cologne Germany; ^4^ Department of Community Medicine, Information and Health Decision Sciences; Faculty of Medicine University of Porto Porto Portugal; ^5^ Knowledge Institute of the Dutch Association of Medical Specialists Utrecht the Netherlands; ^6^ Cochrane Planetary Health Group; Department of Pediatrics University of Alberta Edmonton Alberta Canada; ^7^ Evidence Foundation, Cleveland Heights Ohio USA; ^8^ Department of Medicine McMaster University Hamilton Ontario Canada; ^9^ School of Health and Human Performance Dalhousie University Halifax Canada; ^10^ Barcelona Institute of Global Health (ISGlobal) Universitat Pompeu Fabra Spain; ^11^ Centre for evidence‐based veterinary guidelines University of Copenhagen Denmark; ^12^ IVC Evidensia Södra Djursjukuset Sweden; ^13^ Potsdam Institute for Climate Impact Research (PIK) Potsdam Germany; ^14^ Centre for Climate Change and Planetary Health London School of Hygiene & Tropical Medicine London; ^15^ Cochrane Planetary Health Thematic Group, Institute of General Practice Medical Faculty of the Heinrich‐Heine‐University Düsseldorf Germany; ^16^ Institute of Allergology Charité—Universitätsmedizin Berlin, Corporate Member of Freie Universität Berlin; Humboldt‐Universität zu Berlin Berlin Germany; ^17^ Fraunhofer Institute for Translational Medicine and Pharmacology ITMP Immunology and Allergology Berlin Germany; ^18^ Allergic Rhinitis and Its Impact on Asthma Montpellier France; ^19^ Clinical Epidemiology and Research Center (CERC) IRCCS Humanitas Research Hospital & Department of Biomedical Sciences Milan Italy; ^20^ Lakelands Public Health Peterborough Ontario Canada; ^21^ Department of Family Medicine Queens University Kingston Ontario Canada

**Keywords:** health guidelines, health technology assessment, planetary health, workshop

## Abstract

Health systems contribute to environmental degradation while simultaneously being affected by its consequences. Yet, planetary health considerations are largely absent from health guidelines. On November 6–7, 2024, the Planetary Health Expert Workshop convened over 50 experts from the GRADE Planetary Health Project Group at Humanitas University, Italy, to explore methods for embedding planetary health into health guideline development. Through presentations, case studies, and group discussions, participants identified key methodological gaps and proposed processes and tools to advance the integration of planetary health in health guidelines. Case studies highlighted the need to balance clinical effectiveness with long‐term planetary impacts. This workshop demonstrates a strong interest in integrating planetary health considerations into health guidelines, ensuring evidence‐based health recommendations are not only effective for individuals but also reduce planetary boundary transgressions, restoring Earth's natural systems.

## Summary

- Planetary health is largely absent from health guidelines.
- The GRADE Evidence‐to‐Decision (EtD) should be adapted to incorporate planetary health recommendations across the full guideline life cycle.
- New tools are needed to appraise environmental evidence.
- Incorporating planetary health in health guidelines requires a commitment from organizational leadership, expertise on guideline panels, and structured engagement with interest‐holders.

## 1 Introduction

The healthcare sector accounts for about 5% of the greenhouse gas (GHG) emissions globally [1], which contributes to the degradation of our planet, while simultaneously reducing the resilience of the health sector to the impacts of such harmful Earth system changes. The ongoing degradation of Earth's natural systems poses a growing threat to human health. Of the nine planetary boundaries that are considered important for the resilience of Earth's natural systems and planetary health overall, seven have been transgressed [2]. Here, planetary health refers to the health of all living beings on our planet (including humans) and the natural systems on which they depend [3]. Therefore, it is urgent to consider aspects related to planetary health in the contexts of health technology assessment (HTA) and health decision‐making.

Over the last two decades, the Grading of Recommendations, Assessment, Development and Evaluation (GRADE) guidance has adapted to include considerations of patient values, preferences, and health equity. However, these guideline frameworks have yet to integrate planetary health. In fact, there is GRADE guidance for environmental and occupational health, but not for the assessment of the impact of health interventions on our environment.

With these considerations in mind, a GRADE Planetary Health Expert Workshop was held in Milan (Humanitas University) on November 6–7, 2024, with the aim of developing methods for integrating planetary health into health guideline development. This paper reports on the proceedings and outcomes of the Planetary Health Expert Workshop, which brought together over 50 global experts in clinical epidemiology, health research methodology, guideline development, environmental science, health economics, and health policy to identify and develop conceptual, methodological, and practical strategies for integrating planetary health into health guideline methodologies.

## 2 Workshop Program and Structure

The workshop combined plenary presentations, thematic case studies, and small group discussions. On the first day, the sessions revolved around foundational concepts, methods, and their real‐world applications. On the second day, participants engaged in structured framework development and implementation planning. Key findings are provided in Table 1.

**Table 1:** Consensus, gaps, and priority actions.

Areas of consensus	Key challenges	Priority needs
GRADE Evidence‐to‐Decision (EtD) framework adaptable to planetary domains	Sparse and heterogenous evidence base on environmental impacts of health actions	Standardized, replicable integration methods within GRADE‐based guidelines
Integration must span the full guideline lifecycle (from question formulation to recommendation)	Few explicitly planetary health‐integrated guidelines have been identified	Tools to appraise the certainty of environmental evidence (including LCA studies)
Systems‐level and interdisciplinary collaboration required	No validated approach for rating the certainty of environmental evidence	Core outcome sets combining human health and environmental impact metrics
Planetary health considerations should be embedded upstream in scoping and protocol stages	Methodological variability across environmental impact estimates	Institutional leadership and panel capacity‐building
	Risk of structural deprioritization due to limited expertise and resources	Structured interest‐holder engagement mechanisms

## 3 Foundations for Integrating Planetary Health Into Guideline Methodology

The first day opened with context‐setting remarks and a review of current evidence on integrating planetary health into guidelines. A recurring theme was the recognition that current guidelines and HTA processes do not account for planetary health. A scoping review presented by Thomas Piggott found only four guidelines that explicitly considered planetary health however, the authors of these guidelines had used inconsistent approaches and offered limited methodological transparency [4, 5]. This finding highlights the urgent need to develop standardized and replicable methods for integrating planetary health considerations in guideline development.

Building on this, Alina Hermann proposed a multidimensional framework structured around three clusters for integrating planetary health into health guidelines [6, 7]: (1) describing impacts of planetary transgressions on health; (2) mitigating healthcare's environmental footprint; and (3) implementing strategies that emphasize patient‐centered care and develop quality indicators to measure guideline performance with regard to planetary health.

The discussion then turned to evidence synthesis, which highlighted the methodological challenges of retrieving and evaluating data that span ecological, social, and health domains. Maria Inti‐Metzendorf and Denise Thomson of Cochrane's Planetary Health Thematic Group emphasized the need for interdisciplinary reviews to address cascading risks and their inter‐related outcomes, and logic models to map the complexity of environment‐health interactions [8]. They also presented their work on validated search filters for pathways linking climate change to human health, which are now available [9].

Jonathan Drew presented the open‐access HealthcareLCA database, which has consolidated over 8000 environmental impact estimates from healthcare interventions [10]. Despite the growing evidence base, heterogeneity in methods and poor standardization across various contexts were identified as major limitations [11]. Charlotte Michels then demonstrated how a Dutch surgical guideline panel incorporated planetary health evidence in clinical recommendations in the field of surgery. They integrated the R‐ladder [12, 13], a well‐established sustainability framework that prioritizes circular economy strategies based on environmental efficiency, into their GRADE Evidence‐to‐Decision (EtD) framework to help clinicians identify environmental hotspots and make sustainable choices [13, 14]. In addition, they created a step‐by‐step process for embedding planetary health throughout guideline development.

Klaas Miersch presented on how the climate science community and the Intergovernmental Panel on Climate Change (IPCC) approach the assessment of uncertainty. To inform health guidelines, the effects of additional greenhouse gas emissions on the earth's climate must be translated into societal outcomes to quantify impacts on human wellbeing and to understand how to mitigate these impacts. This requires the assessment of uncertainty for metrics such as the social costs of carbon and the effectiveness of interventions related to climate mitigation and adaptation [15]. Addressing a related theme, Bernardo Sousa‐Pinto and Rafael José Vieira proposed a novel carbon‐utility modeling approach to compare interventions in terms of their carbon impacts while simultaneously considering their effectiveness [16]. This approach is similar to a cost‐effectiveness analysis in that it calculates the incremental environmental cost per unit of health benefit. It could support EtD frameworks by offering a structured, quantitative way to weigh the environmental impact of health interventions against their corresponding effectiveness.

Contributions from One Health and Pathfinder initiatives underscored the importance of adopting systems‐level thinking. Karolina Anna Scahill highlighted that while One Health has historically been associated with zoonoses, updated definitions recognize that it needs to encompass humans, animals, and the whole ecosystem, as captured by the definition of planetary health [17, 18]. Guidelines involving animals or animal products require a holistic view, considering not only GHG emissions but also other factors that the agriculture industry impacts, including but not limited to biodiversity loss, animal welfare, and antimicrobial resistance [19]. Lorna Benton argued that prevention‐focused health guidelines should support systemic changes that address shared social and ecological drivers of poor health [20] and degrading planetary boundaries, with attention to context, inclusion, and equity [21].

Several presenters addressed the role of the GRADE framework in operationalizing planetary health consideration for guidelines methodologies. Emily Senerth noted that the GRADE EtD framework already operationalizes key environmental health concepts across several criteria [22]. For instance, the precautionary principle is reflected in judgments about the priority of the problem and the balance of effects, while environmental justice is considered when looking at social, political, and economic contexts through the criteria of equity, values, acceptability, and feasibility.

Finally, Holger Schünemann emphasized GRADE's potential as a flexible tool that could be adapted to prioritize planetary health impacts, while acknowledging challenges in balancing immediate health outcomes with long‐term environmental impacts.

In the afternoon, a series of case studies was presented showcasing how specific applications of planetary health can be operationalized within specific clinical and public health guideline scenarios [23]. Each case study presented methodological challenges resulting from the need to consider both environmental and human health outcomes.

## 4 Operationalizing Planetary Health in Guideline Development

The second day focused on implementing planetary health considerations in guideline development, including refinement of the GRADE framework to include a planetary health criterion.

Participants proposed criteria for determining when planetary health considerations should be formally integrated, such as when interventions affect high‐prevalence conditions, have high GHG emissions, or where lower‐impact alternatives are available to mitigate environmental impacts. To support this, participants recommended developing a checklist extension to operationalize the integration of planetary health in health guidelines.

Participants discussed the integration of planetary health considerations across the entire guideline development lifecycle, including question formulation, outcome prioritization, and evidence appraisal. Since guidelines involve the formulation and prioritization of questions, having guideline questions centered on planetary health was proposed to incorporate planetary boundaries, such as biodiversity, climate change, and freshwater use, into the construction of guideline questions. This would ensure that guidelines address degrading natural earth systems directly. In addition, participants also discussed the identification of outcomes relevant to planetary health. While the formulation of questions and identification of outcomes relevant for planetary health have some merits, there is a risk that these questions and outcomes may end up not being prioritized by guideline panels due to limited resources and planetary health expertise needed to assess the evidence. In the meantime, GRADE Guidance on integrating planetary health has been prepared, with the manuscript under submission.

The discussions also emphasized the need for expanding the types of evidence considered in guideline development. Given the complexity and limited quantitative data available in environmental health, participants advocated for the inclusion of relevant evidence from secondary data (e.g., infodemiology data), traditional knowledge, qualitative studies, and stakeholder expertise, especially in contexts of uncertainty. The limitations of current methods for assessing the certainty of environmental evidence were also discussed. Proposed solutions included adapting existing GRADE domains in the context of environmental outcomes, and developing new tools for assessing the quality of studies providing environmental data, such as life cycle assessment studies.

Participants also explored the ethical and value‐laden challenges of weighing human versus planetary health outcomes. These discussions recognized that environmental harms often fall on future generations or distant populations, raising questions of intergenerational equity and global justice. One proposed solution was to develop core outcome sets that integrate both human health and environmental impact metrics to ensure balanced recommendations.

The workshop ended with a discussion on preliminary survey findings on planetary health integration in health guidelines, where participants identified opportunities to overcome challenges in its operationalization. In summary, there was a shared recognition that implementing planetary health concepts in guidelines would require not only methodological innovation but also institutional commitment, interest‐holder engagement, and leadership.

## 5 Gaps and Priority Needs

**Methodological gaps:** Currently, no standardized and replicable methods exist for integrating planetary health into guideline development frameworks. Our scoping review found that few guidelines explicitly address planetary health, and even when they do, it is done inconsistently and with limited methodological rigor. Tools for assessing environmental evidence are sparse, e.g., no validated or standardized tools exist to assess the quality of evidence in LCAs. The heterogeneity of environmental impact data across settings further complicates evidence synthesis and comparability, as highlighted by HealthcareLCA.

**Evidence gaps:** Evidence base linking health interventions to planetary outcomes remains thin. Where data do exist, they are fragmented across ecological, social, and clinical domains in ways that current systematic reviews are not well equipped to handle. Metzendorf and Thomson highlighted the need for interdisciplinary reviews and logic models capable of capturing interrelated environment‐health pathways. Core outcome sets that integrate both human health and environmental impact metrics do not yet exist, making it difficult to guide guideline panels to compare these outcomes in a structured manner.

**Institutional and governance gaps:** Because planetary health is a nascent field, guideline panels frequently lack the expertise needed to assess and incorporate relevant evidence. Moreover, resource constraints and other competing priorities mean that planetary health may be deprioritized. Broader institutional commitment and leadership from guideline‐producing organizations are therefore required for meaningful progress.

## 6 Research Plans

The workshop laid out the plan for several future outputs committed to advancing methods and tools to support the integration of planetary health into health guideline development based on the abovementioned gaps. Some of the key research projects for the future included:

1. A glossary and taxonomy paper to clarify terminology, in collaboration with the GRADE ontology group;
2. A GRADE guidance paper outlining what to do for planetary health integration in health guidelines;
3. A GIN‐McMaster checklist extension outlining how to operationalize the GRADE guidance mentioned above;
4. A primer for clinicians to translate planetary health guidance into clinical practice;
5. A chapter on planetary health in the GRADE book; and
6. A commentary advocating for the integration of planetary health considerations into health guidelines [24].

## 7 Conclusions

The Planetary Health Expert Workshop marked an important step toward embedding environmental sustainability within health guideline development. In the 2‐day workshop, participants identified key methodological gaps, explored real‐world applications, and advanced practical tools to integrate planetary health considerations into the GRADE framework.

As health systems confront the realities of climate change, biodiversity loss, and resource depletion, with an additional planetary boundary exceeded in 2025, evidence‐based guidelines must evolve to reflect planetary health as a whole. Collaboration across disciplines, institutions, and geographies is essential to ensure that future health guidelines are equitable, inclusive, just, and environmentally sustainable.

## Author Contributions

**Pakeezah Saadat, Grigorios Leontiadis, Alina Herrmann**, and **Thomas Piggott:** conceptualization. **Pakeezah Saadat:** writing – original draft, methodology, writing – review and editing, investigation. **Maria‐Inti Metzendorf:** writing – original draft, writing – review and editing. **Jean Bousquet:** writing – original draft, writing – review and editing. All authors contributed to the critical revision for important intellectual content and drafting of the work.

## Conflicts of Interest

Alina Herrmann, member of the NGO German Alliance on Climate Change and Health (KLUG e.V.), presents non‐financial conflicts of interest. Holger Schünemann is a co‐chair of the GRADE working group. The rest of the authors declare that they have no conflicts of interest.

## Final Approval

Pakeezah Saadat and Thomas Piggott are accountable for the integrity of the work. All authors approved the final manuscript.

## Data Availability

Data sharing is not applicable to this article as no datasets were generated or analyzed during the current study.
